# A comparison of meta-analytic methods for synthesizing evidence from explanatory and pragmatic trials

**DOI:** 10.1186/s13643-017-0668-3

**Published:** 2018-01-25

**Authors:** Tolulope T. Sajobi, Guowei Li, Oluwagbohunmi Awosoga, Meng Wang, Bijoy K. Menon, Michael D. Hill, Lehana Thabane

**Affiliations:** 1 Department of Community Health Sciences and O’Brien Institute for Public Health, University of Calgary, 3280 Hospital Drive NW, Calgary, Alberta T2N 4Z6 Canada; 2 Department of Health Research Methods, Evidence, and Impact, McMaster University, Hamilton, Ontario Canada; 3 Faculty of Health Sciences, University of Lethbridge, Lethbridge, Alberta Canada; 4 Department of Clinical Neurosciences, University of Calgary, Calgary, Alberta Canada; 5 Department of Clinical Neurosciences and Hotchkiss Brain Institute, University of Calgary, Calgary, Alberta Canada; 6 Biostatistics Unit, Research Institute at St Joseph’s Healthcare—Hamilton, Hamilton, ON Canada

**Keywords:** Meta-analysis, PRECIS-2, Obesity interventions, Randomized controlled trials, Systematic review

## Abstract

**Background:**

The pragmatic–explanatory continuum indicator summary version 2 (PRECIS-2) tool has recently been developed to classify randomized clinical trials (RCTs) as pragmatic or explanatory based on their design characteristics. Given that treatment effects in explanatory trials may be greater than those obtained in pragmatic trials, conventional meta-analytic approaches may not accurately account for the heterogeneity among the studies and may result in biased treatment effect estimates. This study investigates if the incorporation of PRECIS-2 classification of published trials can improve the estimation of overall intervention effects in meta-analysis.

**Methods:**

Using data from 31 published trials of intervention aimed at reducing obesity in children, we evaluated the utility of incorporating PRECIS-2 ratings of published trials into meta-analysis of intervention effects in clinical trials. Specifically, we compared random-effects meta-analysis, stratified meta-analysis, random-effects meta-regression, and mixture random-effects meta-regression methods for estimating overall pooled intervention effects.

**Results:**

Our analyses revealed that mixture meta-regression models that incorporate PRECIS-2 classification as covariate resulted in a larger pooled effect size (ES) estimate (ES = − 1.01, 95%CI = [− 1.52, − 0.43]) than conventional random-effects meta-analysis (ES = − 0.15, 95%CI = [− 0.23, − 0.08]).

**Conclusions:**

In addition to the original intent of PRECIS-2 tool of aiding researchers in their choice of trial design, PRECIS-2 tool is useful for explaining between study variations in systematic review and meta-analysis of published trials. We recommend that researchers adopt mixture meta-regression methods when synthesizing evidence from explanatory and pragmatic trials.

## Background

Randomized controlled trials (RCTs) are cited as the highest level of evidence that can inform clinical and policy decisions about the efficacy and/or effectiveness of an intervention [1–3]. However, RCTs are generally costly, with many stringent inclusion and exclusion criteria which limit generalizability of results and relevance to routine clinical practice. Consequently, there is increased interest in designing RCTs that show real-world effectiveness of an intervention in broad patient populations [4–7]. Schwartz and Lellouch [8] proposed a distinction between explanatory trials, which confirm a physiological or clinical hypothesis, and pragmatic trials, which inform a clinical or policy decision by providing evidence for adoption of the intervention into real-world clinical practice. Since their seminal paper, several papers have investigated the strengths and limitations of pragmatic trials [4–12]. Thorpe et al. [13, 14] proposed the original PRECIS (pragmatic–explanatory continuum indicator summary) tool that further clarified the concept and features of pragmatism and a scoring system and visual representation of the graphical representation of the pragmatic features of a trial. Loudon et al. [15, 16] later proposed a revision of the PRECIS, called PRECIS-2, a 9-item tool to assess the characteristics of a pragmatic design. Features of the PRECIS-2 tools include the recruitment of investigators and participants, the intervention and its delivery, follow-up, and the determination and analysis of outcomes. Many trials could be deemed to be pragmatic with regard to at least one of these dimensions, but few are truly pragmatic on all dimensions.

A number of studies have explored the use of PRECIS instruments when synthesizing evidence from published trials. For example, Patsopoulos [17] suggests that “systematic reviews and meta-analyses could incorporate a PRECIS score for synthesized trials and help the systematic mapping of the pragmatism in published research”. Yoong et al. [18] investigated the impact of pragmatic–explanatory study design characterization on conclusions of systematic reviews of public health interventions of obesity trials. They observed that there were no differences among the intervention effects across classifications of the synthesized studies based on PRECIS ratings. Koppenaal et al. [19] applied a modified version of PRECIS, called PRECIS review tool, to judge the applicability of studies in systematic reviews for daily clinical practice in two systematic reviews [19]. Tosh et al. [20] proposed the pragmascope, an adapted version of the PRECIS tool that uses a 5-point scale to assess the degree of pragmatism when designing RCTs in mental health. Witt et al. [21] conducted a systematic analysis in trials of acupuncture for lower back pain with the intention of applying the PRECIS tool. Glasgow et al. [22] also used the PRECIS tool to describe the design features of three effectiveness trials investigating weight loss in obese patients with comorbid conditions. More recently, Jordan et al. [23] also demonstrated the potential benefit of using the PRECIS-2 instrument for aiding systematic review and meta-analysis of studies in hepatitis C virus care. Louma et al. [24] used PRECIS-2 to identify interventions that effectively increased physical activity and glycemic controls among patients with type 2 diabetes and assess the potential use of PRECIS-2 for implementing physical activity interventions in clinical practice settings.

While the uptake of PRECIS instruments (i.e., PRECIS and PRECIS-2) in systematic reviews is increasing, these instruments are mostly used descriptively but their impact in explaining heterogeneity in meta-analytic investigations has not been investigated. Given the variations in study designs, differences in study characteristics of explanatory and pragmatic trials are likely to influence both statistical heterogeneity and intervention effect estimates in meta-analyses. Aves et al. [25] argues that “….If heterogeneity is substantial, due to 66the degree of pragmatism, it might not be appropriate to pool data from pragmatic and explanatory trials….” Although modern meta-analytic methods such as mixture meta-regression and robust meta-analytic methods have been developed to pool evidence from heterogeneous populations [26–28], there is limited application of these methods and incorporation of PRECIS ratings in synthesizing evidence from explanatory and pragmatic trials.

This study aimed to assess whether the incorporation of PRECIS classification could improve the modeling of heterogeneity among published trials in meta-analytic investigations. Using data from a Cochrane systematic review of 31 trials of community-based obesity intervention in children [29], we compared the performance of random-effects, stratified random-effects, and mixture random-effects meta-regression techniques that accounted for differences between explanatory and pragmatic trials for synthesizing evidence from published trials.

## Methods

### The pragmatic–explanatory continuum indicator summary (PRECIS-2)

PRECIS was developed by a group of international researchers and methodologists to assist trialists in distinguishing between pragmatic and explanatory trial designs [13, 14]. PRECIS requires trialists to indicate on a visual scale (in the shape of wheel) where a trial falls along the pragmatic–explanatory continuum. More recently, a revision of the PRECIS tool, PRECIS-2, was developed [15]. This consists of nine domains, including eligibility, recruitment, setting, organization, flexibility in intervention delivery, flexibility in adherence, follow-up, primary outcome, and primary analysis. Each domain is rated on a 5-point Likert scale from 1 (completely explanatory) to 5 (completely pragmatic) [16].

### Systematic review of obesity prevention trials

Data were from the Cochrane systematic review of trials that investigated the efficacy or effectiveness of community-based obesity prevention interventions in children [29]. The systematic review included all RCTs published between 1990 and March 2010. Similar to the previous work by Yoong et al. [18], we used an adapted version of the PRECIS-2 tool to conduct an audit of all 30 trials of children age 6–12 years included in the Cochrane review of obesity trials to assess the pragmatic–explanatory design features of these studies.

### Raters and rating procedures

Before rating the trials in this systematic review, three study co-authors (TTS, OA, MW) first read and discussed relevant papers on PRECIS [13–16] and piloted their knowledge of the PRECIS tool using 5 randomly selected published trials. Raters then independently rated each of the 31 trials on the 9 domains of the PRECIS-2 tools. Each domain was a score on a 5-point scale that range from 1 (completely explanatory) to 5 (completely pragmatic) using the broad definitions provided by the tool developers [15, 16]. Authors then met to discuss variations in scoring and reached a consensus where there were discrepancies. For each investigator and each trial, an overall summary score was derived by averaging of the ratings of the 9 items. High scores indicated a more pragmatic trial, while lower scores indicated the trial is more explanatory. Since no cut-off scores were provided by the original authors, we applied a scoring method for categorizing the trials as explanatory or pragmatic. Specifically, we classified a trial as explanatory if the average score for the trial is less than 3.0, while a trial is considered pragmatic if the average PRECIS-2 is at least 3.0.

### Statistical analyses

Descriptive statistics was used to summarize the average domain-specific and overall PRECIS-2 scores across the 31 studies included in this analysis. Fleiss kappa statistic was used to assess inter-rater reliability among the domain-specific and overall ratings of each trial on the PRECIS-2 scale [30]. Four meta-analytic methods were used to assess the changes in conclusions about overall intervention effect on body mass index of these children. These include (i) the conventional random-effects meta-analysis; (ii) stratified random-effects meta-analysis, in which effect sizes from pragmatic trials and explanatory trials were independently pooled; (iii) random-effects meta-regression that adjusted for PRECIS-2 rating (explanatory vs pragmatic); and (iv) a mixture random-effects meta-regression that adjusted for PRECIS-2 rating (pragmatic vs explanatory). For each model, we report pooled effect size (ES), 95% confidence interval, between-study variance (τ^2^), and Bayesian information criterion. All analyses, including kappa estimated, were conducted using R software [31].

## Results

**Fig. 1:**
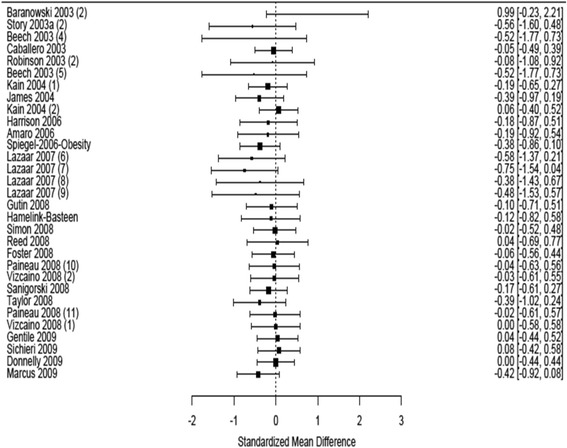
Forest plot obesity trials of children aged 6–12

**Fig. 2:**
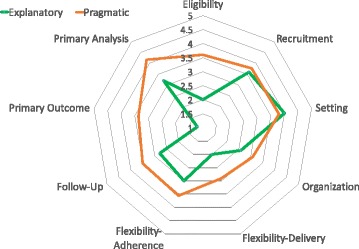
Average PRECIS-2 wheel domain scores for pragmatic and explanatory trial classification of 31 obesity trials in children aged 6–12 years

Of the 31 studies included in our analysis, 12 trials were focused on physical activity interventions only, 5 focused on dietary interventions only, while 14 adopted a combination of physical and dietary interventions [29]. As reported in the Cochrane review [29], the reported standardized mean difference in body mass index between the intervention to reduce obesity in children and the controls for these 31 trials ranged between − 0.36 and 0.45 (Fig. 1). The inter-rater reliability among our independent raters, as measured by the Fleiss kappa, ranged between 0.41 and 0.86, indicating moderate to substantial agreement across the domains [30]. We achieved moderate to substantial agreement for all the domains, with flexibility (delivery) and follow-up domains showing lower agreement (*κ* = 0.41 and 0.48, respectively). The average overall ratings ranged between 2.44 and 4.56. Using a cut-off of 3.0, five studies were classified as explanatory while the remaining 26 studies were classified as pragmatic (see Table 1 and Fig. 2).

**Table 1:** PRECIS-2 ratings and characteristics of 31 published trials of interventions to reduce obesity in children aged 6–12 years

Study	Type of treatment	Standardized mean difference	Standard error^*^	Average overall PRECIS rating	PRECIS classification
Baranowski 2003 (2)	Physical activity and dietary	0.99	0.388	2.44	Explanatory
Lazaar 2007 (6)	Physical activity	− 0.58	0.16	2.78	Explanatory
Lazaar 2007 (7)	Physical activity	− 0.75	0.16	2.78	Explanatory
Lazaar 2007 (8)	Physical activity	− 0.38	0.29	2.78	Explanatory
Lazaar 2007 (9)	Physical activity	− 0.48	0.29	2.78	Explanatory
Story 2003a (2)	Physical activity and dietary	− 0.56	0.28	3.22	Pragmatic
Beech 2003 (4)	Physical activity and dietary	− 0.52	0.41	3.11	Pragmatic
Caballero 2003	Physical activity and dietary	− 0.05	0.05	3.00	Pragmatic
Robinson 2003 (2)	Physical activity and dietary	− 0.08	0.26	3.00	Pragmatic
Beech 2003 (5)	Physical activity and dietary	− 0.52	0.41	3.11	Pragmatic
Kain 2004 (1)	Physical activity and dietary	− 0.19	0.06	3.33	Pragmatic
James 2004	Dietary	− 0.39	0.09	4.50	Pragmatic
Kain 2004 (2)	Physical activity and dietary	0.06	0.06	3.33	Pragmatic
Harrison 2006	Physical activity	− 0.18	0.12	3.89	Pragmatic
Amaro 2006	Dietary	− 0.19	0.14	3.11	Pragmatic
Spiegel-2006-Obesity	Physical activity and dietary	− 0.38	0.06	3.56	Pragmatic
Gutin 2008	Physical activity	− 0.10	0.10	3.67	Pragmatic
Hamelink-Basteen	Physical activity and dietary	− 0.12	0.13	3.0	Pragmatic
Simon 2008	Physical activity	− 0.02	0.07	4.33	Pragmatic
Reed 2008	Physical activity	0.04	0.14	3.89	Pragmatic
Foster 2008	Physical activity	− 0.06	0.07	3.78	Pragmatic
Paineau 2008 (10)	Dietary	− 0.04^*^	0.09	3.00	Pragmatic
Vizcaino 2008 (2)	Physical activity	− 0.03^*^	0.09	3.00	Pragmatic
Sanigorski 2008	Physical activity and dietary	− 0.17	0.05	4.25	Pragmatic
Taylor 2008	Physical activity and dietary	− 0.39	0.10	3.00	Pragmatic
Paineau 2008 (11)	Dietary	− 0.02	0.09	3.00	Pragmatic
Vizcaino 2008 (1)	Physical activity	0.00	0.09	3.00	Pragmatic
Gentile 2009	Physical activity and dietary	0.04	0.06	4.00	Pragmatic
Sichieri 2009	Dietary	0.08	0.07	4.56	Pragmatic
Donnelly 2009	Physical activity	0.00	0.05	4.22	Pragmatic
Marcus 2009	Physical activity and dietary	− 0.42	0.07	3.25	Pragmatic

*Rounded up to the nearest two decimal places

**Fig. 3:**
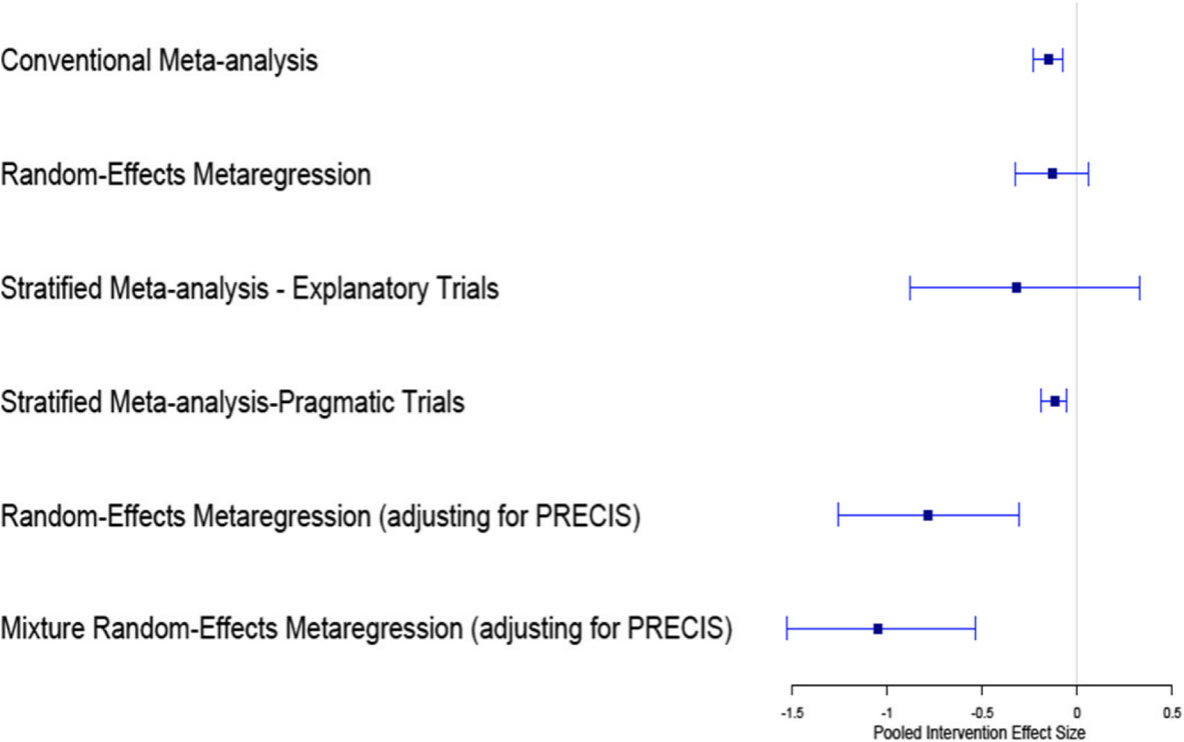
Comparison of meta-analytic methods for estimating overall intervention effect in trials for reducing obesity in children aged 6–12 years

Table 2 and Fig. 3 describe the estimates of pooled intervention effect size based on random-effects meta-regression methods for 31 published trials. Conventional pooling of the intervention effects across all the studies based on random-effects meta-analysis suggest statistically significant pooled ES of − 0.15 (95%CI = [− 0.23, − 0.08]). When we fitted stratified meta-analysis independently for pragmatic and explanatory trials, the meta-analysis of the explanatory trials revealed no significant pooled intervention effect for the explanatory trials (ES = − 0.32; 95%CI = [− 0.88, 0.33]) but statistically significant pooled intervention effect for the pragmatic trials (ES = − 0.12; 95%CI = [− 0.19, − 0.06]). Nevertheless, the pooled intervention effect from the explanatory trials was about 2.5 times the pooled effect size obtained from the pragmatic trials. Meta-regression methods that adjusted for overall PRECIS-2 ratings revealed significantly larger pooled effect sizes and smaller *τ*^2^ than the conventional overall pooled effect size. Specifically, the random-effects meta regression model that adjusted for PRECIS-2 rating revealed a statistically significant pooled effect size (ES = − 0.79, 95%CI = [− 1.26, − 0.31] that is more than five times larger than the estimated pooled effect size obtained from the conventional meta-analysis model. The mixture random-effects meta-regression model that controlled for PRECIS rating (pragmatic vs explanatory) even revealed substantially large and statistically significant pooled effect size (ES = − 1.05, 95%CI = [− 1.53, − 0.54]) (Fig. 3).

**Table 2:** Comparison of meta-analytic methods for estimating overall intervention effect in trials to reduce obesity in children aged 6–12 years

Meta-analytic methods	Pooled effect size	95%CI	*τ* ^2^	BIC
Random-effects meta-analysis^a^	− 0.15	[− 0.23, − 0.08]^*^	0.029	8.08
Stratified meta-analysis				
Explanatory trials^b^	− 0.32	[− 0.88, 0.33]	0.209	− 11.97
Pragmatic trials^b^	− 0.12	[− 0.19, − 0.06]^*^	0.018	− 9.74
Meta-regression methods				
REMR controlling for PRECIS	− 0.79	[− 1.26, − 0.29]^*^	0.020	− 5.33
Mixture REMR controlling for PRECIS	− 1.01	[− 1.52, − 0.43]^*^	0.010	− 10.39

*τ*^2^ between-trial variance, *BIC* Bayesian information criterion, *REMR* random effects mixture regression, *95%CI* 95% confidence interval

^*^p < 0.05

^a^Unadjusted model

## Discussion

The incorporation of PRECIS-2 classification of the trials as covariate in meta-regression models results in larger estimates of pooled intervention effects in published trials than previously reported pooled effect sizes [19, 29]. Meta-regression methods, such as mixture regression models that controls for PRECIS-2 rating as a covariate, are particularly advantageous in that they can account for heterogeneity among the studies by modeling the heterogeneity attributable to pragmatism using mixture distribution. This finding supports results from previous research that recognize the importance of accounting for between-study heterogeneity that is attributable to the degree of pragmatism in systematic review of published trials [18, 27]. It highlights the utility of PRECIS-2 tool for aiding synthesis of evidence from published trials and the impact of information that design characteristics can have in explaining the between-study heterogeneity when conducting meta-analysis of published studies. We recommend that researchers should not only use PRECIS-2 rating information descriptively in meta-analysis of published studies but also for inferential purposes for modeling heterogeneity when estimating pooled intervention effects.

Also, we found that stratified meta-analytic methods, in which explanatory and pragmatic trials are defined with explicit criteria and independently synthesized, supported the notion that there was an overall significant effect of the community-based obesity intervention in children in pragmatic trials but not in explanatory trials. The estimated pooled effect size obtained from synthesis of pragmatic trials was significantly smaller than the estimated pooled effect size obtained from synthesis of explanatory trials. This finding is in line with previous research that shows explanatory trials often report larger effect sizes than pragmatic trials [18]. One main advantage of stratified meta-regression methodology is that it can help researchers and policy makers understand the strength of evidence for efficacy and/or effectiveness of an intervention in a population. It can also aid policy decision making about an intervention when the direction of pooled effect size in both pragmatic and explanatory trials are in the same direction. However, policy decision making about an intervention based on this stratified meta-analysis approach may not always be straightforward especially when the estimated pooled intervention effect sizes in both explanatory and pragmatic trials are in opposite directions. Few studies have recommended that policy decision making should be based on evidence from pragmatic trials only since they confirm the real-world effectiveness of an intervention [23, 24, 32]. But this recommendation may not be valid when there is limited number of pragmatic trials included in the systematic review due to low statistical power.

Our comparisons of the meta-analytic methods for synthesizing evidence from published trials rely on observed data only. Future research will explore the use of Monte Carlo methods to examine the statistical properties of these methods including their statistical power, bias, mean square error, and coverage under a variety of data analytic conditions. Importantly, the accuracy of the pooled intervention effects obtained from meta-regression analysis hinges on the accuracy of the PRECIS-2 ratings to assess the degree of pragmatism in each trial. While the ratings obtained from the three reviewers in our study had good overall inter-rater agreement, flexibility delivery and follow-up domains of PRECIS-2 exhibited only moderate agreement. This is consistent with previous studies that report high variability or poor agreement on flexibility and/or follow-up domains [18, 19, 23, 24]. Additionally, we had missing scores on some of the PRECIS domains for some studies because the original articles lack this information. Although Yoong et al. [18] recommend that investigators should endeavor to contact primary authors of each published trial with incomplete information when using PRECIS-2, we did not contact authors of the original articles but derived the mean scores on PRECIS domains and overall score based on all the available scores [18, 19]. Future research will use sensitivity analysis to assess the impact of missing data on estimates of pooled effect sizes from meta-analytic investigations. Moreover, while we have analyzed the overall PRECIS-2 scores for these published trials, the component domains that constitute this overall score may have ratings that vary on the explanatory–pragmatic continuum. The PRECIS domain-specific information about these trials might provide policy makers with relevant information (e.g., about implementation of interventions).

## Conclusion

This study shows that the incorporation of information about the type of trials (explanatory or pragmatic), assessed using PRECIS-2 tool, can influence the estimation of pooled intervention effects in meta-analysis of published trials when there is substantial heterogeneity attributable to pragmatism. Secondly, this study also reveals the need for meta-regression methods that, adjusts for PRECIS information as covariate, for estimating pooled intervention effects in meta-analytic investigations. This ensures that valid conclusions are derived from systematic reviews and meta-analysis of published trials. We recommend that meta-analytic investigations in systematic reviews should incorporate information about design characteristics using PRECIS-2 when synthesizing evidence from published studies.

## Abbreviations

95%CI: 95% confidence interval
ES: Effect size
PRECIS-2: Pragmatic–explanatory continuum indicator summary version 2
RCT: Randomized controlled trial
SD: Standard deviation
